# Phytosulfokine peptides, their receptors, and functions

**DOI:** 10.3389/fpls.2023.1326964

**Published:** 2024-01-05

**Authors:** Yi Li, Qi Di, Li Luo, Liangliang Yu

**Affiliations:** ^1^ Shanghai Key Laboratory of Bio-energy Crops, School of Life Sciences, Shanghai University, Shanghai, China; ^2^ State Key Laboratory of Subtropical Silviculture, Zhejiang A&F University, Hangzhou, Zhejiang, China

**Keywords:** cell division, cell growth, immunity, phytosulfokine, PSKR, sulfated peptide

## Abstract

Phytosulfokines (PSKs) are a class of disulfated pentapeptides and are regarded as plant peptide hormones. PSK-α, -γ, -δ, and -ϵ are four bioactive PSKs that are reported to have roles in plant growth, development, and immunity. In this review, we summarize recent advances in PSK biosynthesis, signaling, and function. PSKs are encoded by precursor genes that are widespread in higher plants. PSKs maturation from these precursors requires a sulfation step, which is catalyzed by a tyrosylprotein sulfotransferase, as well as proteolytic cleavage by subtilisin serine proteases. PSK signaling is mediated by plasma membrane-localized receptors PSKRs that belong to the leucine-rich repeat receptor-like kinase family. Moreover, multiple biological functions can be attributed to PSKs, including promoting cell division and cell growth, regulating plant reproduction, inducing somatic embryogenesis, enhancing legume nodulation, and regulating plant resistance to biotic and abiotic stress. Finally, we propose several research directions in this field. This review provides important insights into PSKs that will facilitate biotechnological development and PSK application in agriculture.

## Introduction

Plant growth and development are regulated by intrinsic and extrinsic signals. Phytohormones represent several kinds of important intrinsic signaling molecules that regulate plant growth, reproduction, and adaptation to biotic and abiotic stress. After systemin, an 18 amino acids long polypeptide, was first isolated from tomato (*Solanum lycopersicum*) leaves (Pearce et al., 1991), dozens of other signaling polypeptides have been discovered in the plant kingdom. In contrast to traditional phytohormones, which are small metabolite molecules, these polypeptides have amino acid backbones that can include post-translational modifications, such as tyrosine sulfation, proline hydroxylation, etc. Similarly to traditional phytohormones, polypeptide signaling molecules regulate various aspects of plant growth and development; moreover, they are typically regarded as peptide hormones because of their ability to function at very low concentrations (Matsubayashi and Sakagami, 2006).

Among the peptide hormones, phytosulfokines (PSKs) belong to a group of pentapeptide hormones with tyrosine sulfation modifications. The first PSK was identified as a proliferation inducer of *Asparagus officinalis* mesophyll cells that were cultured at low density (Matsubayashi and Sakagami, 1996). Over the past nearly three decades, studies have revealed that PSKs not only induce *in vitro* cell division, but can also promote cell growth in various plant organs, regulate plant reproduction, induce somatic embryogenesis, enhance root nodule formation in legumes, and regulate responses to biotic and abiotic stress, among other functions. This review focuses on the current understanding of PSK peptides and precursors, PSK formation, receptor-mediated signaling, and their biological functions.

## PSK peptides and their precursors

**Figure 1:**
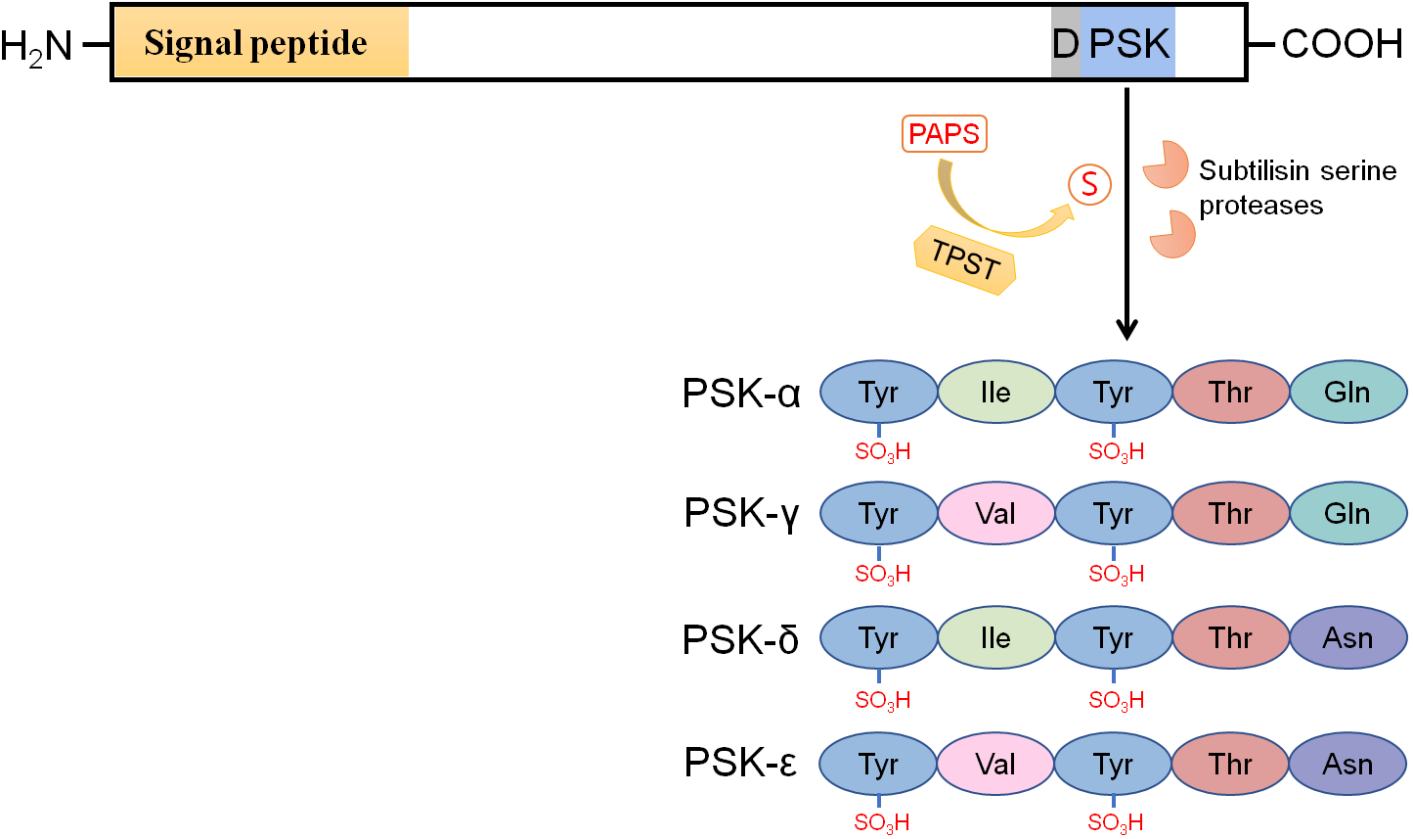
PSK precursors have an N-terminal signal peptide for targeting the secretory pathway and a PSK peptide sequence located near their C-termini. The aspartic acid residue preceding the PSK peptide sequence is highly conserved. Tyrosine sulfation of PSKs, including PSK-α, -γ, -δ, and -ϵ, is catalyzed by tyrosylprotein sulfotransferases (TPSTs) in the *cis*-Golgi apparatus with 3’-phosphoadenosine 5’-phosphosulfate (PAPS) as the sulfate group donor. After secretion into the apoplast, the sulfated PSK precursors are cleaved by subtilisin serine proteases (AtSBT1.1, AtSBT3.8, SlPhyt2, etc.) to release the mature PSK peptides. The PSK-α peptide widely exists in spermatophytes, while PSK -γ, -δ, and -ϵ peptides are specifically encoded by legume species.

Proliferation of plant cells in culture strictly depends on cell density. Plant cells at low density culture usually have low mitotic activity and cannot be induced by treatment with known phytohormones. However, cell proliferation can be promoted by supplementation of conditioned medium prepared from rapidly growing cells in culture (Yang et al., 2000). To isolate the key mitogenic factor(s) from the conditioned medium, Matsubayashi and Sakagami (1996) developed a sensitive bioassay using *Asparagus officinalis* mesophyll cell cultures at a low density. Finally, they successfully identified a mitogenic factor, which was a disulfated pentapeptide with the sequence Y_SO3_IY_SO3_TQ, and named it PSK-α (**Figure 1**). When measuring PSK-α activity, a disulfated tetrapeptide (PSK-β) with the sequence Y_SO3_IY_SO3_T was also co-purified (Matsubayashi and Sakagami, 1996). Compared with PSK-α, the PSK-β peptide had a much lower biological activity (8% activity relative to PSK-α; Matsubayashi et al., 1996), and hence, it is now reclassified as a degradation product of PSK-α (Yang et al., 1999). Recently, three novel types of PSK peptides (PSK-γ, -δ, and -ϵ) were identified from various legume species. PSK-γ with the sequence Y_SO3_VY_SO3_TQ (**Figure 1**) is distinguished from PSK-α by a valine in the second amino acid position. The PSK-γ-encoding precursor genes *GmPSKγ1* and -*2* are primarily expressed in the developing seeds of soybean (*Glycine max*), and ectopic expression of *GmPSKγ1* significantly improves seed cell growth (Yu et al., 2019). PSK-δ (Y_SO3_IY_SO3_TN, **Figure 1**), which differs from PSK-α at the last amino acid position, is another type of PSK peptide. PSK-δ precursor genes are highly expressed in legume root nodules, and the mature PSK-δ peptide has also been detected by LC-MS in *Medicago truncatula* root nodules (Yu et al., 2022). The last member of the PSK family is PSK-ϵ (Y_SO3_VY_SO3_TN, **Figure 1**) with two amino acid differences from PSK-α. Both PSK-δ and PSK-ϵ positively regulate root nodule formation in legume species (Di et al., 2022; Yu et al., 2022).

PSK peptides are generated from 80 to 120 amino acids long precursors (Yang et al., 2001; Lorbiecke and Sauter, 2002). These precursors have an N-terminal signal peptide for targeting the secretory pathway and a PSK pentapeptide sequence located close to their C-termini (**Figure 1**; Yang et al., 2000; Sauter, 2015). PSK precursors are encoded by gene families that are ubiquitous in spermatophytes, including angiosperms and gymnosperms (**Table 1**). No PSK member has been reported or identified through homologous search in basal plants, indicating that PSK peptides exist specifically in higher plants. The PSK gene family has seven members in Arabidopsis (Stuhrwohldt et al., 2021), eight in tomato (Zhang et al., 2018), ten in pear (Kou et al., 2020), and seven in *M. truncatula* (Di et al., 2022). These PSK members are putatively functional orthologues across different species. Although the PSK peptide sequence and aspartic acid residue preceding the PSK peptide sequence are highly conserved (**Figure 1**), PSK precursors share little similarity, both within and across plant species, at the amino acid level (Lorbiecke and Sauter, 2002; Yu et al., 2022).

**Table 1:** Functionally characterized PSK precursor genes.

Plant species	Name	Function	Reference(s)
Arabidopsis (*Arabidopsis thaliana*)	*AtPSK1*	Promotes root growth, improves tolerance to drought stress	(Kutschmar et al., 2009; Stuhrwohldt et al., 2021)
	*AtPSK2*	Controls pollen tube growth and funicular pollen tube guidance, regulates immune response against pathogens	(Mosher et al., 2013; Rodiuc et al., 2015; Stuhrwohldt et al., 2015)
	*AtPSK4*	Promotes root and leaf growth, promotes callus formation, induces male sterility, regulates immune response against pathogens	(Matsubayashi et al., 2006; Mosher et al., 2013; Rodiuc et al., 2015; Yu et al., 2016)
	*AtPSK5*	Regulates cell division in root quiescent center	(Heyman et al., 2013)
Rice (*Oryza sativa*)	*OsPSK*	Promotes cell proliferation and callus formation	(Yang et al., 1999; Yang et al., 2000)
Maize (*Zea mays*)	*ZmPSK1*, *ZmPSK3*	Regulates reproduction	(Lorbiecke et al., 2005)
Zinnia (*Zinnia elegans*)	*ZePSK1*	Stimulates tracheary element differentiation	(Matsubayashi et al., 1999; Motose et al., 2009)
Cotton (*Gossypium hirsutum*)	*GhPSK*	Promotes cotton fiber elongation	(Han et al., 2014)
	*lncRNA7* (encoding PSK-α)	Enhances cotton resistance to Verticillium wilt	(Zhang et al., 2022)
Pear (*Pyrus bretschneideri*)	*PbrPSK2*	Promotes pollen tube growth	(Kou et al., 2020)
Tomato (*Solanum lycopersicus*)	*SlPSK1*, *SlPSK6*	Promotes drought-induced flower drop	(Reichardt et al., 2020)
	*SlPSK3*, *SlPSK3L*	Enhances immunity against *Botrytis cinerea*	(Zhang et al., 2018)
Wheat (*Triticum aestivum*)	*TaPSK5*	Promotes root growth and yield traits	(Geng et al., 2020)
*Cryptomeria japonica*	*CjPSK1*	Promotes somatic cell embryogenesis	(Igasaki et al., 2003)
Chinese fir (*Cunninghamia lanceolata*)	*ClPSK*	Promotes somatic cell embryogenesis	(Hao et al., 2022)
Rubber tree (*Hevea brasiliensis*)	*HbPSK5*	Promotes laticifer formation to increase rubber content	(Chao et al., 2023)
*Lotus japonicus*	*LjPSK1*	Promotes root growth and nodule formation	(Wang C, et al., 2015)
	*LjPSKδ*	Promotes root nodule formation	(Yu et al., 2022)
Soybean (*Glycine max*)	*GmPSKγ1*	Promotes seed growth	(Liu et al., 2019; Yu et al., 2019)
*Medicago truncatula*	*MtPSKδ*	Promotes root nodule formation	(Yu et al., 2022)
	*MtPSKϵ*	Promotes root elongation, lateral root formation, and nodulation	(Di et al., 2022)

## PSK processing and maturation

PSK precursors require tyrosine sulfation and proteolytic cleavage to generate mature bioactive PSK peptides (**Figure 1**). Unsulfated PSK has previously been reported to promote root growth to a lesser degree than sulfated PSK, thereby indicating the essential role of tyrosine sulfation for full bioactivity in PSKs (Kutschmar et al., 2009; Yu et al., 2016). PSK tyrosine sulfation is catalyzed by plant-specific tyrosylprotein sulfotransferases (TPSTs) with 3’-phosphoadenosine 5’-phosphosulfate (PAPS) as the sulfate group donor (**Figure 1**, Hanai et al., 2000a). TPST activity is initially detected in the microsomal membrane fractions of cells in several plant species, and the conserved aspartic acid residue at the -1 position of the PSK peptides is essential for the sulfation reaction (Hanai et al., 2000a). In Arabidopsis, AtTPST, which is localized in the *cis*-Golgi apparatus, is encoded by a single copy gene, *AtTPST*, that is widely expressed in various tissues. (Komori et al., 2009). The loss-of-function mutant of *AtTPST* displays a severe dwarf phenotype accompanied by stunted roots (Komori et al., 2009), which indicates the importance of PSK and/or other sulfated peptides in plant growth and development.

The sulfated PSK precursors are subsequently secreted into the apoplast where they are proteolytically cleaved to release the mature PSK peptides. The apoplast-localized subtilisin serine protease (AtSBT1.1) in Arabidopsis is reported to cleave proAtPSK4, leaving three residues at the N-terminus of the PSK pentapeptide (Srivastava et al., 2008). The presence of these N-terminus residues suggests that an additional exopeptidase is required to generate mature PSKs. Moreover, AtSBT1.1 exhibits specificity for proAtPSK4 and displays little or no activity with other AtPSK members in Arabidopsis (Srivastava et al., 2008), thereby suggesting that more proteases are involved in PSK maturation within the plant. Recently, Stuhrwohldt et al. (2021) have reported another subtilisin serine protease (AtSBT3.8) that cleaves the AtPSK1 precursor. Interestingly, AtSBT3.8 can only cleave at the C-terminus of the PSK peptide sequence, and it is highly specific to the AtPSK1 precursor, given that it is the only precursor with an aspartic acid immediately downstream of the PSK peptide sequence. Despite ongoing work, the complexity of the mechanisms by which PSK peptides are released from their precursors in Arabidopsis means that the process is still far from being understood. In tomato, PSK maturation is processed by SlPhyt2, a phytaspase subtype of the subtilisin-like protease (Reichardt et al., 2020). SlPhyt2 cleaves precursors at the N-terminus cleavage site of the PSK peptide sequence and requires the conserved aspartic acid preceding the PSK peptide sequence for substrate recognition and cleavage.

## PSK receptors and signal transduction

**Figure 2:**
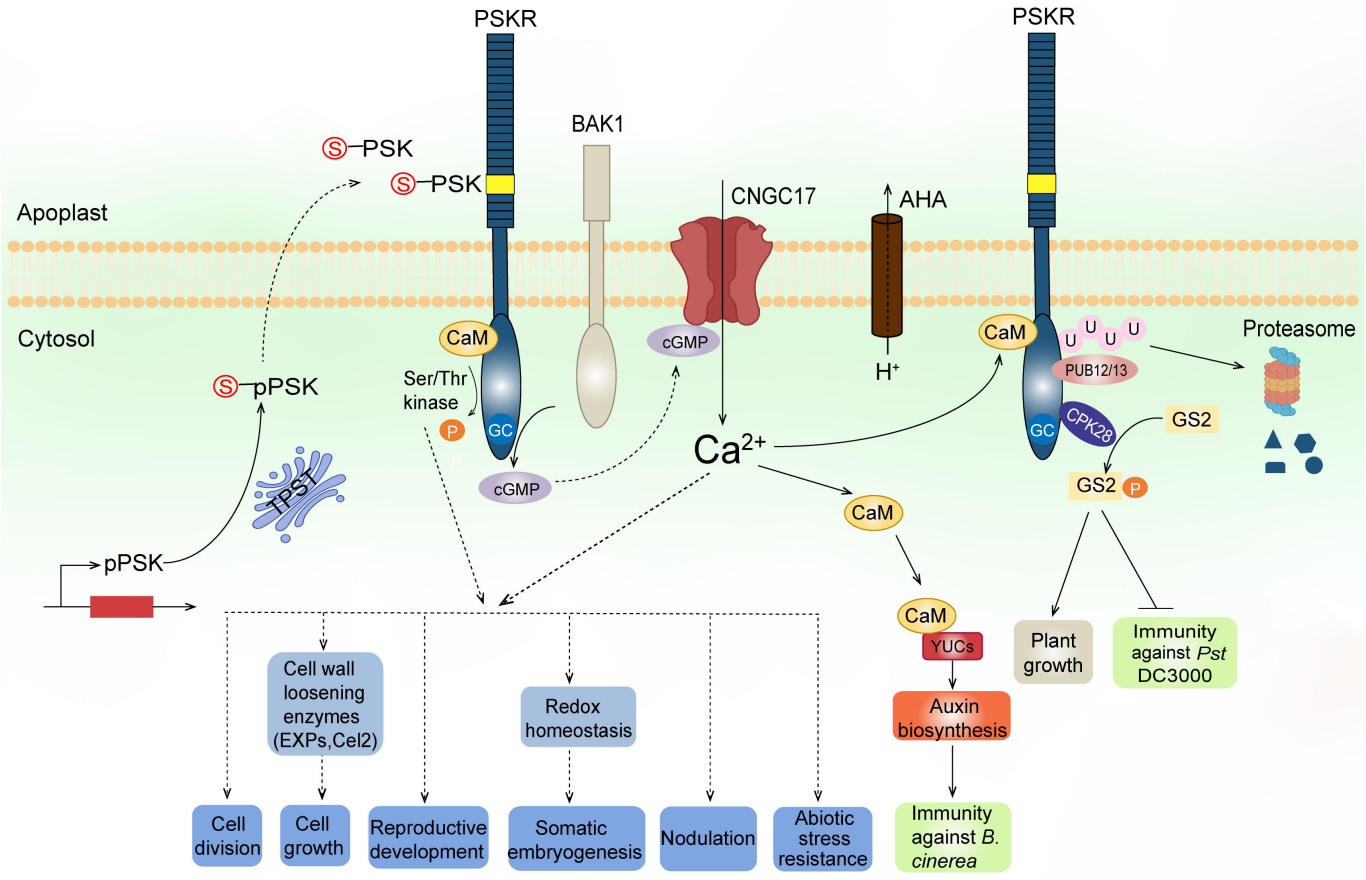
A working model of PSK biosynthesis, signaling, and functions. PSK precursors (pPSKs) undergo tyrosine sulfation (indicated by red S) catalyzed by a TPST in the *cis*-Golgi followed by proteolytic cleavage in the apoplast. The mature PSK peptides are perceived by their plasma membrane-localized receptors, PSKRs. The PSKRs have an extracellular domain containing 21 LRRs (blue bars) and an island domain (yellow box) to which PSKs bind, a transmembrane domain, and a cytoplasmic domain. The cytoplasmic domain has a Ser/Thr kinase probably regulated by CaM binding and an overlapping GC activity that produces cGMP. PSKR, its co-receptor BAK1, AHAs, and cGMP-activated CNGC17 form a functional module to link proton extrusion to Ca^2+^ influx across the plasma membrane. The increased cytosolic Ca^2+^ signal is further transduced to CaMs which bind to auxin biosynthetic proteins YUCs, leading to auxin-mediated immunity against *Botrytis cinerea*. The PSKR interacts with CPK28, which in turn phosphorylates GS2 at different sites to inversely regulate growth and immunity against *Pst* DC3000. Furthermore, E3 ligases PUB12/13 interact with the PSKR and cause ubiquitylation to degrade the receptor, maintaining a tight control of PSKR abundance at the posttranslational level. The kinase activity of PSKRs and/or the generated Ca^2+^ signaling predictably regulate downstream elements to confer biological functions, including regulation of cell division, cell growth, reproduction, somatic embryogenesis, legume nodulation, and resistance to abiotic stress.

PSK peptides bind to the plasma membrane-localized receptors (PSKRs), which belong to the X subclass of the leucine-rich repeat receptor-like kinase (LRR-RLK) family (Matsubayashi et al., 2002; Morillo and Tax, 2006). The first PSKR (DcPSKR1) was purified from carrot microsomal fractions using ligand-based affinity chromatography (Matsubayashi et al., 2002). The interaction between PSK and DcPSKR1 is highly specific and tight, with low nanomolar dissociation constants. DcPSKR1 consists of an N-terminal signal peptide that targets the receptor to the secretary pathway, an extracellular domain containing 21 LRRs, a transmembrane domain, and a cytoplasmic kinase domain (**Figure 2**, Matsubayashi et al., 2002). LRRs 17 and 18 are separated by an island domain that is composed of 36 amino acids and is rich in hydrophilic and charged amino acid residues (Matsubayashi et al., 2002). Photoaffinity labeling assays reveal that a 15 amino acid region (Glu503-Lys517) in the island domain of DcPSKR1 is responsible for PSK binding (Shinohara et al., 2007). Crystal structure studies further demonstrate that PSK interacts mainly with the β-strand of the PSKR island domain, where it stabilizes the island domain for recruitment of co-receptor(s) (Wang J, et al., 2015).

Based on the amino acid similarity with DcPSKR1, two PSKRs in Arabidopsis (AtPSKR1 and AtPSKR2; Matsubayashi et al., 2006; Amano et al., 2007), and two in tomato (Zhang et al., 2018) were also later discovered (**Table 2**). AtPSKR1 has a similar structure as DcPSKR1 (Matsubayashi et al., 2006) and was confirmed by fluorescence microscopy of the AtPSKR1-GFP fusion protein to be localized at the plasma membrane (Hartmann et al., 2014; Rodiuc et al., 2015). The *AtPSKR1* gene shows a ubiquitous expression pattern in all Arabidopsis organs (Matsubayashi et al., 2006; Kutschmar et al., 2009), while *AtPSKR2* expression is only detected in flowers (Stuhrwohldt et al., 2015), which is indicative of a major role of AtPSKR1 in PSK signal transduction in Arabidopsis. The legume species encode PSK -γ, -δ, and -ϵ peptides as well as PSK-α (Yu et al., 2019; Di et al., 2022; Yu et al., 2022). However, only one receptor gene *MtPSKR*, which is ubiquitously expressed, has been identified in *M. truncatula*, suggesting that all the PSK peptides are recognized by the same receptor.

**Table 2:** Functionally characterized PSK receptor genes.

Plant species	Name	Function	Reference(s)
Carrot (*Daucus carota*)	*DcPSKR1*	Promotes callus growth	(Matsubayashi et al., 2002)
Arabidopsis (*Arabidopsis thaliana*)	*AtPSKR1*	Promotes plant growth, promotes callus formation, controls pollen tube growth and funicular pollen tube guidance, regulates immune response against pathogens	(Matsubayashi et al., 2006; Kutschmar et al., 2009; Stuhrwohldt et al., 2011; Igarashi et al., 2012; Mosher et al., 2013; Rodiuc et al., 2015; Stuhrwohldt et al., 2015)
	*AtPSKR2*	Controls pollen tube growth and funicular pollen tube guidance	(Stuhrwohldt et al., 2015)
Rice (*Oryza sativa*)	*OsPSKR1*	Regulates immune response against bacterial leaf streak	(Yang et al., 2019)
	*OsPSKR15*	Enhances drought stress tolerance	(Nagar et al., 2022)
Tomato (*Solanum lycopersicus*)	*SlPSKR1*	Promotes plant growth, regulates immune response against pathogens	(Zhang et al., 2018; Ding et al., 2022; Hu et al., 2023)

PSK receptors appear to have both kinase and guanylate cyclase (GC) activities (**Figure 2**). Expression of the cytoplasmic domain of AtPSKR1 shows that it is an active Ser/Thr kinase with both autophosphorylation and transphosphorylation activities (Kwezi et al., 2011; Hartmann et al., 2014). It has been found that S696 and S698 in the juxtamembrane domain of PSKR1 are phosphorylated, and that site-directed mutations at these sites result in reduced transphosphorylation activity of the kinase and subsequent impaired shoot growth (Kaufmann et al., 2017). Moreover, site-directed mutation of T998 at the C-terminus region abolishes PSKR1 kinase activity but not the receptor function (Kaufmann et al., 2017). In addition, AtPSKR1 possesses a calmodulin (CaM) binding site within the kinase domain, at which all four Arabidopsis CaM isoforms can be bound to the receptor (Hartmann et al., 2014). Site-directed mutation assays show that Ca^2+^/CaM binding is required for AtPSKR1 kinase activity and, consequently, necessary for PSK signaling to promote growth (Hartmann et al., 2014). In addition, it has been found that AtPSKR1 contains a putative GC catalytic core embedded within the kinase domain (Kwezi et al., 2011). The kinase domain expressed *in vitro* also exhibits GC activity. Overexpression of the full-length AtPSKR1 receptor in Arabidopsis leaf protoplasts dramatically raises the endogenous cyclic GMP (cGMP) levels, and this effect is further enhanced after PSK-α application, thereby indicating that PSKR1 has a GC activity *in vivo* (Kwezi et al., 2011).

Crystal structure analysis reveals that PSK binding stabilizes the PSKR island domain for recruitment of a somatic embryogenesis receptor-like kinase (SERK), such as SERK1, SERK2, or BAK1 (Wang J, et al., 2015). BAK1 physically interacts with PSKR1 in Arabidopsis, and *BAK1* loss-of-function mutants do not respond to PSK, thereby indicating that BAK1, or other SERKs, may be functioning as co-receptor(s) of PSKR1 (Ladwig et al., 2015). Besides BAK1, the H^+^-ATPases AHA1 and AHA2 have also been shown to interact with PSKR1. BAK1 and AHAs in turn bind to the cyclic nucleotide-gated cation channel CNGC17, which is required for PSK-induced cell expansion (Ladwig et al., 2015). CNGCs are known targets of cGMP in plant cells (Talke et al., 2003). Protoplast expansion assay using the *cngc17* mutant reveals that cGMP functions in a CNGC17-dependent manner (Ladwig et al., 2015). As mentioned above, AtPSKR1 has a GC center which produces cGMP *in vivo*. Mutating the GC center impairs Arabidopsis growth, thereby supporting the role of cGMP in PSKR1 signaling *in planta* (Ladwig et al., 2015). Based on these findings, it is conceivable that PSKRs, activated by PSK binding, produce cGMP, which positively regulates CNGC17 activity. The activated CNGC17 forms a functional module with BAK1 and AHAs to link proton extrusion to cation (like Ca^2+^) uptake across the plasma membrane, leading to enhanced cell growth (**Figure 2**). It is worth noting that the cGMP-mediated CNGC17 signaling mechanism was reported in PSK-regulated cell growth. Whether the same pathway or direct regulation of downstream elements by cGMP, as a second messenger, exists in other physiological processes needs further study.

In tomato, PSK detection by the main receptor SlPSKR1 increases cytosolic Ca^2+^ concentrations and leads to auxin-mediated immune responses against *Botrytis cinerea* by promoting binding activity between CaMs and the auxin biosynthetic proteins YUCCA (YUCs) (**Figure 2**, Zhang et al., 2018). Yeast two-hybrid screening provides evidence that the calcium-dependent protein kinase CPK28 interacts with SlPSKR1. CPK28, in turn, phosphorylates the glutamine synthetase GS2, which then regulates plant growth and immunity (**Figure 2**, Ding et al., 2022). More recently, the plant U-box E3 ligases PUB12/13 were found to interact with SlPSKR1 and cause SlPSKR1 ubiquitylation at the Lys-748 and Lys-905 positions to degrade this receptor, and thereby maintain a tight control of PSKR abundance at the posttranslational level (**Figure 2**, Hu et al., 2023).

## PSK functions

### PSK promotes plant cell division

Plant cells cultured at low density usually have low mitotic activity which can be induced by addition of a conditioned medium. PSK-α was originally isolated from the conditioned medium of asparagus mesophyll cell cultures, and it has been shown to act as a strong inducer of plant cell division at nanomolar concentrations in suspension culture (Matsubayashi and Sakagami, 1996; Matsubayashi et al., 1997). Moreover, many plant tissues and cells dedifferentiate and re-divide to form calli under special culture conditions. For instance, in rice cell cultures, expression of the sense *OsPSK* sequence significantly promotes cell proliferation to form large calli, while the *OsPSK*-antisense cells generate small ones (Yang et al., 1999). Similarly, in Arabidopsis, PSK-AtPSKR1 signaling is also shown to induce explant cell division and form larger calli (Matsubayashi et al., 2002; Matsubayashi et al., 2006). Thus, these findings demonstrate that PSK peptides promote plant cell division *in vitro*.

Furthermore, the quiescent center (QC) plays a key role in plant root development by preserving the stem cell state in the cells surrounding the QC (van den Berg et al., 1997). Heyman et al. (2013) reported that the ERF115 transcription factor acts as a rate-limiting factor in QC cell division by transcriptionally activating the *AtPSK5* gene, thereby resulting in enhanced PSK-PSKR signaling, which in turn promotes QC cell division. Thus, this *in vivo* effect that PSK has on the QC effectively ensures the longevity of the root stem cells even when surrounding stem cells become damaged (Heyman et al., 2013).

### PSK promotes plant cell growth

PSKs and their receptors have been extensively reported to positively regulate the growth of various plant organs and tissues, which is in agreement with their ubiquitous expression patterns during plant growth and development (Matsubayashi et al., 2006; Kutschmar et al., 2009). Moreover, PSKs significantly promote root elongation (Matsubayashi et al., 2006; Kutschmar et al., 2009; Wang C, et al., 2015; Yu et al., 2019; Yu et al., 2020), hypocotyl elongation (Stuhrwohldt et al., 2011), and leaf growth (Matsubayashi et al., 2006; Yu et al., 2016). Such profound effects of PSK signaling on plant growth are primarily attributed to enlargement in cell size rather than an increase in cell proliferation. Application of synthetic PSK-α peptides in Arabidopsis markedly promotes cell elongation in both roots and hypocotyls. Conversely, loss-of-function mutants of *AtPSKR1* and *AtTPST* both develop short organs with decreased cell size, providing genetic evidence to support PSKs’ function in promoting cell growth (Matsubayashi et al., 2006; Kutschmar et al., 2009; Stuhrwohldt et al., 2011). Furthermore, treatment with PSK-α peptides or overexpression of a PSK precursor gene, *GhPSK*, promotes cotton (*Gossypium hirsutum*) fiber cell elongation, thereby producing longer and finer fibers (Han et al., 2014).

It is well known that plant cell growth requires two aspects of cellular alteration: cell wall loosening and protoplast expansion driven by intracellular turgor pressure. PSKs appear to regulate both of these events during induced cell growth. Stuhrwohldt et al. (2011) reports that treatment with PSK-α peptides induces protoplast expansion in a dose-dependent manner (Stuhrwohldt et al., 2011). However, cells *in planta* are surrounded by a primary cell wall structure composed of cellulose, hemicellulose, and pectin, which form an intricate network (Cosgrove, 2005). Therefore, cell wall remodeling and loosening is the crucial first step before cell expansion can occur (Chebli and Geitmann, 2017). A recent transcriptomics study on Arabidopsis *TPST* mutant seedlings that were treated with PSK-α peptides reveals that many genes related to cell wall modification, organization, or biogenesis — including pectin, galacturonan, and carbohydrate metabolism — are differentially regulated by PSK signaling (Kaufmann et al., 2021). Indeed, several cell wall loosening enzymes, including expansins and endo-1,4-β-glucanases, act downstream of PSK signaling to regulate cell growth. For instance, three expansin genes, *AtEXPA2*, ***-*** *11*, and -*15*, are transcriptionally activated in *AtPSK4*-overexpressing Arabidopsis (Yu et al., 2016), and an endo-1,4-β-glucanase-encoding gene *Cel2* is up-regulated as a result of *GmPSKγ1* overexpression (Yu et al., 2019). Conversely, expression of two *Pectin Methylesterase Inhibitor* (*PMEI*) genes, which repress cell wall loosening, are down-regulated after PSK-α treatment in *M. truncatula* roots (Yu et al., 2020).

### PSK regulates plant reproductive development

Pollen germination and tube growth *in vitro* are typically dependent on pollen density, and this phenomenon is known as the pollen population effect (Pasonen and Kapyla, 1998). Adding PSK-α peptides to a low-density culture stimulates pollen germination and tube growth (Chen et al., 2000; Stuhrwohldt et al., 2015; Kou et al., 2020), thereby indicating that PSK peptide is the key inducer that is involved in the pollen population effect. Furthermore, some PSK precursor and receptor genes show organ- or tissue-specific expression patterns during reproduction, including in flowers and seeds, which is suggestive of important roles of PSK signaling in reproductive development *in planta*. *AtPSK2* in Arabidopsis (Stuhrwohldt et al., 2015), *ZmPSK1* in maize (Lorbiecke et al., 2005), and *PbrPSK2* in pear (Kou et al., 2020) are all highly expressed in the pollen, while transcripts of *ZmPSK1* and *ZmPSK3* are also detected in the egg and central cells of the female gametophyte in maize (Lorbiecke et al., 2005). Moreover, the Arabidopsis PSK receptor double mutant *pskr1pskr2* and *TPST* mutant, which block PSK signaling and maturation, respectively, both display reduced fertility, which is mainly attributed to the loss of funicular pollen tube guidance from the transmitting tract to the embryo sac (Stuhrwohldt et al., 2015). Intriguingly, excess PSK peptide production via *AtPSK4* overexpression also induces sterility, thereby resulting in the failure in fibrous cell wall thickening in the anther’s endothecium (Yu et al., 2016). Thus, results from these studies suggest that a normal PSK level is especially important for plant reproduction. Furthermore, *GmPSKγ1* is specifically expressed in the developing seeds of soybean, and overexpression of this gene significantly promotes seed growth by inducing embryo cell expansion (Yu et al., 2019). Similarly, overexpression of *TaPSK5*, a wheat (*Triticum aestivum*) PSK precursor gene, significantly increases grain size and number in rice (Geng et al., 2020). Taken together, accumulating evidence strongly suggests that PSK peptides function as important regulators in pollen germination and tube growth, guidance of pollen tubes to the embryo sac, development of the anther endothecium layer, and seed growth.

### PSK stimulates somatic embryogenesis and regeneration

Plant somatic embryogenesis (SE) refers to the developmental process of embryos originating from somatic cells without fusion of gametes. SE is an ideal procedure not only for efficient production of clonal plant stocks, but also for targeting tissues for genetic transformation. PSK has been found to stimulate SE in the carrot (*Daucus carota*) and *Cryptomeria japonica* (Hanai et al., 2000b; Igasaki et al., 2003), and *C. japonica* seedlings regenerated via SE grow normally (Igasaki et al., 2003). In contrast, legume species are generally known for their recalcitrance to regeneration approaches. However, PSK treatment dramatically increases the *in vitro* embryogenic and organogenic regeneration competence, thereby subsequently producing regenerated legume plants (Ochatt et al., 2018). Recently, Hao et al. (2022) also reported that PSK treatment remarkably increases SE efficiency in — and even establishes SE in recalcitrant genotypes of — *Cunninghamia lanceolata*, a woody conifer species, by maintaining redox homeostasis in *C. lanceolata*. Collectively, these findings show that PSK signaling has stimulatory effects on somatic embryogenesis and regeneration and that PSK peptides have promising uses in plant genetic transformation and molecular breeding programs.

### PSK promotes nodulation in legumes

Symbiotic interactions of legumes with soil *Rhizobia* species result in the formation of root nodules that fix atmospheric dinitrogen into ammonia, which in turn supports host plant growth (Oldroyd et al., 2011). Nitrogen-fixing nodule development requires the coordination of rhizobial infection at the legume root epidermis and cell re-division in the cortex, the latter of which is a process known as nodule organogenesis (Yang et al., 2022). In *Lotus japonicus*, which is a common model legume species, five PSK-α precursor genes have been identified. Among them, *LjPSK1* and *LjPSK4* exhibit a root nodule-specific expression pattern. Both treatment with the PSK-α peptide and overexpression of *LjPSK1* enhance nodulation by promoting nodule maturation (Wang C, et al., 2015). Recently, Yu et al. (2022) also identified a novel type of PSK, PSK-δ, and its precursor genes in various legume species, including *M. truncatula*, *L. japonicus*, and *G. max*. Using LC-MS on *M. truncatula*, the disulfated PSK-δ peptide is mainly detected in the nodules. Moreover, promoter-GUS assays show that its precursor gene *MtPSKδ* is expressed during the entire nodule developmental process, which also includes nodule organogenesis in the root cortex. Both overexpression of *MtPSKδ* and treatment with the PSK-δ peptides promote symbiotic nodulation in legumes mainly by enhancing nodule organogenesis. Genetic evidence further demonstrates that PSK-δ and PSK-α function redundantly in promoting nodule organogenesis (Yu et al., 2022). In addition, another new type of PSK, PSK-ϵ, has also been found to promote nodulation as well as enhance root elongation and lateral root formation in *M. truncatula* (Di et al., 2022). Collectively, accumulating evidence indicates that different types of PSK peptides, including PSK-α, PSK-δ, and PSK-ϵ, synergistically promote nodule formation in legumes.

### PSK regulates plant immune response

Pattern-triggered immunity (PTI) is the first layer in plant innate immunity and is triggered by the recognition of microbe-associated molecular patterns (MAMPs) (Boller and Felix, 2009). The PSK receptor gene *AtPSKR1* is induced by MAMP treatment in Arabidopsis, and its loss-of-function mutant (*pskr1*) displays an enhanced PTI response against the (hemi)biotrophic bacterial pathogen *Pseudomonas syringae* pv*. tomato* (*Pst*) DC3000 (Igarashi et al., 2012). Consistently, overexpression of either the PSK precursor or receptor genes in Arabidopsis enhances its susceptibility to *Pst* DC3000 (Mosher et al., 2013), thereby indicating that PSK signaling attenuates immune responses against bacterial pathogens. In contrast, the *pskr1* mutant is more susceptible to the necrotrophic fungal pathogen *Alternaria brassicicola*, which provides evidence that PSK increases the plant’s ability to resist necrotrophic pathogen infections (Mosher et al., 2013). Therefore, given these observations, PSK signaling is capable of regulating Arabidopsis’ immune response to biotrophic and necrotrophic pathogens in an antagonistic manner. The antagonistic effect of PSK signaling on different types of pathogens has also been recently verified in tomato. On the one hand, the tomato PSK receptor SlPSKR1 interacts with CPK28, which in turn phosphorylates the glutamine synthetase GS2 at the Serine-334 position in order to repress the resistance against *Pst* DC3000 (Ding et al., 2022); on the other hand, SlPSKR1-mediated PSK signaling increases cytosolic Ca^2+^ concentrations and induces an auxin-mediated immune response against the necrotrophic fungus *B. cinerea* (Zhang et al., 2018). Further research in tomato shows that the U-box E3 ligases PUB12/13 repress SlPSKR1 via ubiquitylation and thus suppresses the PSK-induced immune response against *B. cinerea* (Hu et al., 2023). Furthermore, Rodiuc et al. (2015) reported that Arabidopsis *pskr1* mutants disrupt the development of the feeding structure in oomycetes and root-knot nematodes, as well as their ability to reproduce, after they infect the plant, thereby suggesting that PSK signaling in the host helps these two biotrophic pathogens with establishment and disease development. Recently, PSK signaling has also been revealed to positively regulate resistance to *Verticillium dahlia* via auxin-mediated signaling in cotton (Zhang et al., 2022). Given that PSK regulates plant growth, it is conceivable that PSK signaling acts as an important regulator that integrates the immune response with plant growth so as to balance the two energy-consuming processes.

### PSK regulates resistance to abiotic stress

The PSK-α peptide has been shown to protect plant growth under high night-time temperature conditions, thereby indicating that PSK signaling enhances plant resistance to heat stress (Yamakawa et al., 1999). Expression levels of several PSK precursor genes and of three subtilisin-like serine protease (SBT) genes are up-regulated during osmotic stress in Arabidopsis (Stuhrwohldt et al., 2021). Moreover, overexpression of *AtSBT3.8*, which encodes a protease responsible for PSK-α maturation, or *AtPSK1* increases tolerance to drought stress (Stuhrwohldt et al., 2021). Similarly, overexpression of a rice PSK receptor OsPSKR15 improves the plant’s ability to tolerate drought stress in an abscisic acid-dependent manner (Nagar et al., 2022). In tomato, the expression of *SlPhyt2*, which encodes a subtilisin-like protease to generate mature PSK peptide, is also induced by drought stress. More specifically, when the plant experiences drought stress, the mature peptide acts in the abscission zone of flower pedicels, where it induces the expression of cell wall hydrolases to promote flower abscission (Reichardt et al., 2020). Overall, these findings provide substantial evidence that PSK signaling confers resistance against abiotic stress factors *in planta*.

### Other functions of PSK

PSK-α peptide stimulates tracheary element differentiation in cultured mesophyll cells of zinnia (*Zinnia elegans*) in a dose-dependent manner (Matsubayashi et al., 1999; Motose et al., 2009). In Arabidopsis roots, mutations in the PSK receptors or *TPST* gene result in ectopic xylem differentiation in the procambial cells, which indicates that PSK signaling regulates xylem cell fate by helping to maintain procambial identity (Holzwart et al., 2018). More recently, a study on the rubber tree (*Hevea brasiliensis*) genome identified the PSK-encoding gene *HbPSK5* to be a key gene involved in the domestication of this tree species; in this respect, *HbPSK5* promotes laticifer formation in the rubber tree, which then leads to increased rubber content (Chao et al., 2023).

## Future perspectives

The disulfated PSK pentapeptides play important roles in various growth and developmental processes in plants. Improving our understanding of how PSK peptides are produced from their precursors is of great value. Indeed, relative to the tyrosine sulfation mechanism, the mechanisms by which PSK precursors are proteolytically cleaved to release the bioactive pentapeptides are still far from being understood. In Arabidopsis, subtilisin serine proteases AtSBT1.1 and AtSBT3.8 are reported to cleave AtPSK4 (Srivastava et al., 2008) and AtPSK1 (Stuhrwohldt et al., 2021) precursors, respectively. However, the enzyme activity in these proteases depends on the presence of specific PSK members, without which the pentapeptides cannot be generated. Consequently, further investigation on proteases involved in PSK maturation is needed.

PSK peptides interact with their receptors (PSKRs) to transduce signals. The cytoplasmic domain of a PSKR is found to have dual kinase and GC activities and both processes are required for PSK-PSKR signaling *in planta* (Kwezi et al., 2011; Hartmann et al., 2014; Ladwig et al., 2015; Kaufmann et al., 2017). Whether the PSKR kinase and GC regulate different downstream events or coordinately modulate common developmental events is still unknown. Therefore, detailed dissection of the functions of the PSKR domains and the mechanisms by which their activity is regulated is required to fully understand PSK receptors. To date, little is known about the components that are downstream of the PSK receptors. Hence, the downstream signaling factors, including kinases, phosphatases, transcription factors, and enzymes, await elucidation.

It is clear that PSK signaling-induced plant growth is primarily caused by the cell wall remodeling and cell expansion. Actually, the other aspects that PSKs regulated, such as pollen germination and tube growth, somatic embryogenesis, and root nodule organogenesis, also undergo intense cell expansion. Whether PSKs’ functions in these physiological processes are eventually explained by their activity in cell wall remodeling and cell expansion is worth further investigating.

Lastly, PSK signaling, along with other phytohormones, regulates plant growth, development, and resistance against biotic and abiotic stress. Current reports on PSK interactions with other phytohormones remain very few. Therefore, further investigation on the crosstalk between PSK signaling and other phytohormones, as well as on the underlying regulatory mechanisms, will be crucial to fully elucidate the functions of PSK.

## Author contributions

YL: Writing – original draft. QD: Writing – original draft. LL: Writing – review & editing. LY: Conceptualization, Data curation, Funding acquisition, Supervision, Writing – original draft, Writing – review & editing.
